# The role of metabolic bariatric surgery in improving outcomes for major adverse cardiovascular events

**DOI:** 10.1097/MS9.0000000000003014

**Published:** 2025-02-27

**Authors:** Chao-Ming Hung, Hui-Ming Lee, Chong-Chi Chiu

**Affiliations:** a Department of General Surgery, E-Da Cancer Hospital, I-Shou University, Kaohsiung, Taiwan; b School of Medicine, College of Medicine, I-Shou University, Kaohsiung, Taiwan; c Department of Medical Education and Research, E-Da Cancer Hospital, I-Shou University, Kaohsiung, Taiwan

*Dear Editor*,

Metabolic bariatric surgery, as demonstrated by Pan *et al*, holds the potential to induce significant weight loss and improve metabolic parameters, offering a beacon of hope in the management of diabetes^[1]^. Elevated blood glucose levels can lead to endothelial dysfunction, increased arterial stiffness, and inflammation, all precursors to atherosclerosis and other cardiovascular complications^[2]^. Additionally, diabetes is often associated with other risk factors, such as hypertension and dyslipidemia, further compounding cardiovascular risks^[3]^. Therefore, achieving remission or significant control of diabetes post-surgery can profoundly impact overall cardiovascular health.

Pan *et al* delve into considerations for patient selection and shared decision-making regarding surgical options. This tailored approach reflects an understanding that individual patient profiles can significantly influence surgical outcomes. The authors rightly highlight the critical need for further research into metabolic bariatric surgery’s efficacy and safety profiles, including how factors such as age, sex, socioeconomic status, and comorbidities influence surgical outcomes. This ongoing research is vital for refining patient selection criteria and optimizing surgical techniques, ensuring that all patients receive optimal care based on their unique characteristics, health circumstances, and ongoing compliance.

However, we should consider the long-term safety of one-anastomosis gastric bypass (OAGB) and single-anastomosis duodenal-jejunal bypass with sleeve gastrectomy (SADJB-SG). Although the short- to medium-term results are promising, comprehensive long-term data on potential complications is still lacking^[4]^. Besides, issues of nutritional deficiencies, gastrointestinal complications, and the need for ongoing medical supervision post-surgery are critical factors that must be addressed in future research. Understanding these risks is essential for informed patient consent and ensuring that individuals are fully aware of the potential long-term implications of their surgical choices.

Another point of discussion revolves around the generalizability of the study’s findings. Conducted within a specific population in Taiwan, cultural, genetic, or lifestyle factors may influence outcomes that do not necessarily apply to other demographics. Therefore, additional studies involving diverse populations are needed to validate these results universally. This is particularly important given the varying prevalence of obesity-related comorbidities across different regions and populations.

While the literature presents compelling evidence supporting the efficacy of OAGB and SADJB-SG in promoting weight loss and reducing cardiovascular risks among Taiwanese patients, various perspectives must be considered when interpreting these findings. Proponents advocate for broader implementation of metabolic bariatric surgery as a viable treatment option for obesity; however, skepticism regarding generalizability, long-term safety concerns, and implications for healthcare policy warrant careful consideration. Ultimately, ongoing research and dialogue among healthcare professionals will be essential for advancing our understanding of metabolic bariatric surgery’s role in addressing obesity and its associated health challenges globally.

Lowering the 10-year risk of major adverse cardiovascular events in metabolic bariatric surgery patients requires a comprehensive strategy that encompasses preoperative evaluations, careful selection of surgical techniques, robust postoperative care, adjustment of medical treatment, continuous monitoring of glycemic levels, and ongoing lifestyle modifications. By addressing both the physiological and psychological aspects of obesity management through a multidisciplinary approach, healthcare providers can significantly improve long-term outcomes for these patients. The commitment to continuous monitoring and adaptation of care plans based on individual progress reassures patients of the potential for sustained health benefits following metabolic bariatric surgery.

As obesity continues to pose a substantial burden on healthcare systems worldwide^[5]^, policymakers must consider how best to allocate resources for metabolic bariatric surgery. Proponents argue that investing in these surgical interventions can lead to long-term cost savings by reducing healthcare expenditures associated with obesity-related complications. However, detractors caution that these investments may not yield the desired outcomes without adequate infrastructure—such as access to detailed preoperative education and continuous postoperative follow-up.

As healthcare providers continue to explore practical strategies for managing obesity and its comorbidities, emphasizing the importance of diabetes remission will be critical for enhancing patient outcomes and reducing long-term cardiovascular risks. Ongoing research into optimizing surgical techniques and postoperative care will further refine our understanding of this relationship and improve clinical practices to enhance patients’ quality of life, promote long-term health in bariatric surgery patients, and reduce healthcare costs associated with obesity-related complications. Efforts to increase patient participation in managing obesity should focus on addressing cultural and logistical barriers, enhancing patient education on the benefits of long-term care, and fostering better communication between healthcare providers and patients.

## Data Availability

Not applicable.

## Research registration unique identifying number (UIN)

Not required.
